# Choroid Plexitis and Ependymitis by Magnetic Resonance Imaging are Biomarkers of Neuronal Damage and Inflammation in HIV-negative Cryptococcal Meningoencephalitis

**DOI:** 10.1038/s41598-017-09694-0

**Published:** 2017-08-23

**Authors:** Dima A. Hammoud, Eman Mahdi, Anil A. Panackal, Paul Wakim, Virginia Sheikh, Irini Sereti, Bibi Bielakova, John E. Bennett, Peter R. Williamson

**Affiliations:** 1 Center for Infectious Disease Imaging (CIDI), Radiology and Imaging Sciences, Clinical Center, National Institutes of Health (NIH), Bethesda, MD USA; 2 Division of Pediatric Radiology, Diagnostic Imaging and Radiology, Children’s National Health System, Washington, DC USA; 3 Laboratory of Clinical Infectious Diseases, National Institute of Allergy and Infectious Diseases, National Institutes of Health, Bethesda, Maryland USA; 4 Division of Infectious Diseases, Department of Medicine, F. Hebert School of Medicine, Uniformed Services University of the Health Sciences (USUHS), Bethesda, MD USA; 5 Biostatistics and Clinical Epidemiology Service, Clinical Center, National Institutes of Health (NIH), Bethesda, MD USA; 6 Division of Intramural Research, National Institute of Allergy and Infectious Diseases (NIAID), NIH, Bethesda, MD USA; 7 Neuroimmunological Diseases Unit, Neuroimmunology Branch, National Institute of Neurological Diseases and Stroke (NINDS), NIH, Bethesda, MD USA

## Abstract

CNS cryptococcal meningoencephalitis in both HIV positive (HIV+) and HIV negative (HIV−) subjects is associated with high morbidity and mortality despite optimal antifungal therapy. We thus conducted a detailed analysis of the MR imaging findings in 45 HIV− and 11 HIV+ patients to identify imaging findings associated with refractory disease. Ventricular abnormalities, namely ependymitis and choroid plexitis were seen in HIV− but not in HIV+ subjects. We then correlated the imaging findings in a subset of HIV− subjects (n = 17) to CSF levels of neurofilament light chain (NFL), reflective of axonal damage and sCD27, known to best predict the presence of intrathecal T-cell mediated inflammation. We found that ependymitis on brain MRI was the best predictor of higher log(sCD27) levels and choroid plexitis was the best predictor of higher log(NFL) levels. The availability of predictive imaging biomarkers of inflammation and neurological damage in HIV− subjects with CNS cryptococcosis may help gauge disease severity and guide the therapeutic approach in those patients.

## Introduction

Central nervous system (CNS) cryptococcal meningoencephalitis (CM) remains a common opportunistic infection in HIV positive (HIV+) patients, especially in developing countries with limited access to antiretroviral therapies although its prevalence has been declining in the US and other developed countries^1^. The prevalence of the infection in previously healthy, HIV-negative (HIV−) patients, on the other hand, has been persistent^2^ even in developed countries where it accounts for almost a third of the cases^3^ and is associated with 30% mortality despite optimal therapy^2, 4, 5^. In contrast to HIV+ patients who have defective cell immunity, previously healthy, HIV− patients with CM and clinically refractory disease show a strong intrathecal expansion and activation of innate and adaptive immunity cells despite effective microbiological control. Activated T-cells include primed CD4+ T-cells that recognize cryptococcal antigen and express high levels of IFN-γ, accompanied with increased cerebrospinal fluid (CSF) levels of neurofilament light chain (NFL), reflective of axonal damage^5^.

Assessing responsiveness to immunosuppressive agents including corticosteroids^6^ creates a need for readily available clinical biomarkers of inflammation and neuronal damage that can be used in combination with microbiological biomarkers, such as early fungicidal activity^7, 8^, to individualize and guide therapy. The imaging findings of CM have been described in HIV+^9–15^, and HIV− patients^16–23^, including meningitis, meningoencephalitis, hydrocephalus, isolated cryptococcomas and disseminated cryptococcosis. Additional findings in HIV− subjects include choroid plexitis and ependymitis as well as pachymeningeal enhancement and ischemic infarcts^18, 22, 24–26^. There has been however no direct comparison of imaging findings between the two populations and no attempts at correlating MR imaging findings with CSF indicators of inflammation and lymphocyte activation.

The present studies seek to identify imaging biomarkers that would be most predictive of CSF lymphocytic activation and inflammation patterns in the HIV− population, by correlating MRI findings with cerebrospinal fluid (CSF) levels of NFL and a soluble marker of T-cell activation, sCD27, recently shown to best predict the presence of intrathecal T-cell mediated inflammation compared to other biomarkers^27^. To provide a broader clinical context, we also compared these imaging manifestations to those of HIV+ subjects presenting with CM to better understand differences in disease pathophysiology between the two populations.

## Results

The mean age for the previously healthy HIV− subjects was 49.1 +/− 12.8 years at the time of scanning while the mean age for HIV+ subjects was 39 +/− 9.9 years. In the HIV− group, only four out of 45 patients were on steroids at the time of the scanning (Table 1). Forty one out of 45 patients were on antifungals (either Amphotericin B or Fluconazole). Twenty one patients had negative cryptococcal antigens in the CSF at the time of the scans while 22 had positive antigen titers ranging from 1:2 to 1:1024. No antigen titers were available for two of the HIV− patients. Only two out of 45 patients had positive cultures for *C. neoformans* at the time of the imaging.

**Table 1:** Summary of clinical patient characteristics.

Parameters	HIV− patients with documented CNS cryptococcosis	HIV+ patients with documented CNS cryptococcosis
**N**	45	11
Age (mean ± STD)	49.1 +/−12.8	39.0 +/− 9.9
Gender, Male: Female	15: 30	11: 0
Treatment: Corticosteroids	4 (8.9%)	0 (0%)
Treatment: Antifungal agents	41 (91.1%)	5 (45.5%)
Positive CSF Cryptococcal antigen titers	22 (48.9%)	10 (91%)
CSF Cryptococcal antigen titers range	1:2 to 1:1024	1:2 to 1:1024
Positive CSF cultures for *C. neoformans*	2 (4.4%)	5 (45.5%)
CD4 cell counts (cells/µL)	—	78 +/− 92
HIV viral load range (copies/ml)	—	<50 to 1.10E + 06
Treatment: Antiretroviral therapy	—	5 (45.5%)

In the HIV+ group, 10 out of 11 patients had positive cryptococcal antigens titers in the CSF (range from 1:2 to 1:1024) with no titer measured for one patient (Table 1). Five out of 11 patients had positive CSF cultures for *C. neoformans*, while six had negative cultures. CD4 values ranged from 0 to 327 cells/µL at time of imaging (mean CD4 count = 78 +/− 92 cells/µL) and viral load varied from <50 to 1.1 million copies/ml. Five out of 11 subjects were on Fluconazole treatment and 5 were on antiretroviral therapy.

**Figure 1:**
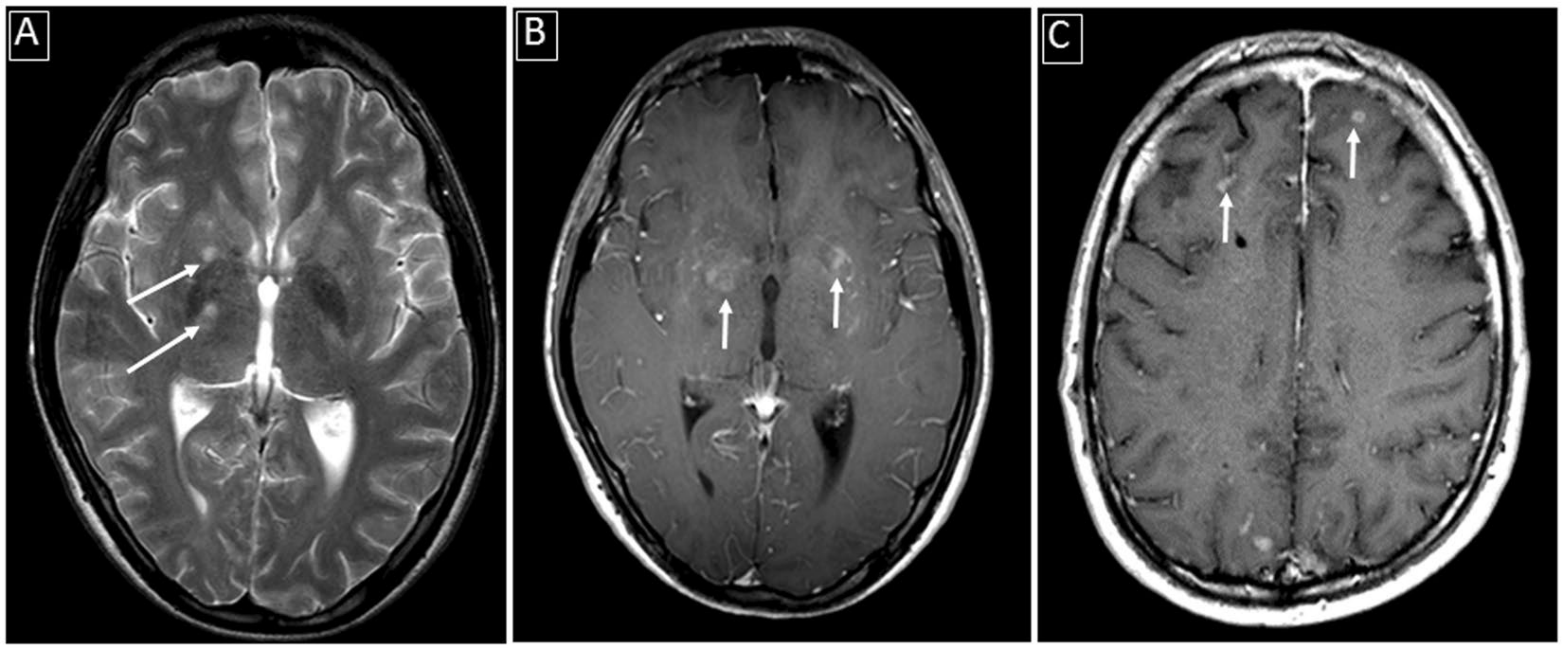
Examples of parenchymal involvement in CNS cryptococcosis: (**A**) Abnormally enlarged perivascular spaces in the basal ganglia bilaterally (white arrows) on T2-weighted image; (**B**) Abnormal enhancing lesions in the basal ganglia (white arrows) in the same patient as A on enhanced T1-weighted image; (**C**) Superficial focal parenchymal enhancing lesions (white arrows) adjacent to sulcal meningeal enhancement.

**Figure 2:**
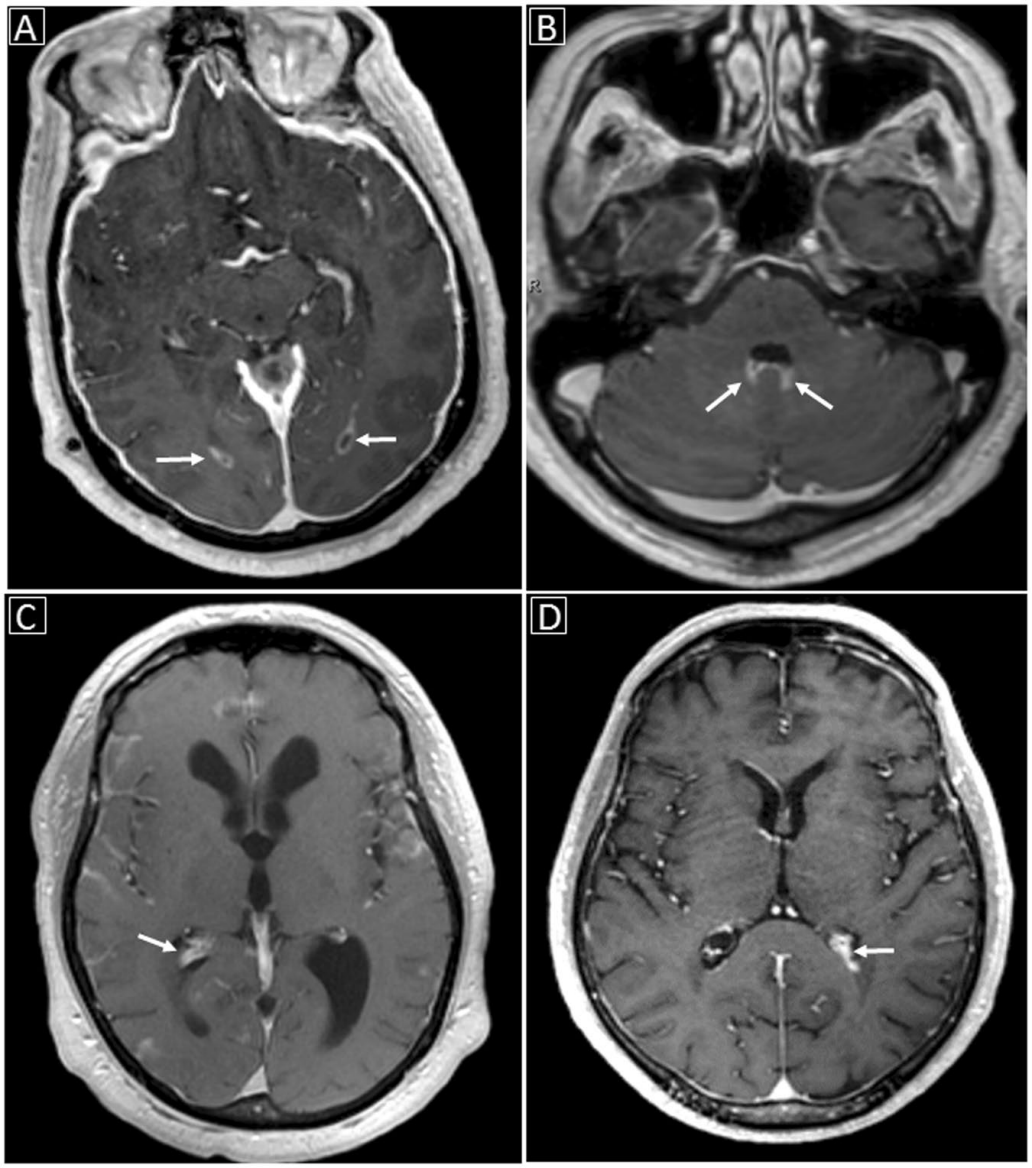
Examples of ventricular involvement in CNS cryptococcosis: (**A**) Abnormal ependymal enhancement along the occipital horns of the lateral ventricles bilaterally (white arrows) on enhanced T1-weighted image; (**B**) Abnormal ependymal enhancement along the posterior aspect of the fourth ventricle with probable associated choroid plexitis (white arrows); (**C**) Choroid plexitis of the right lateral ventricle (white arrow) with hydrocephalus; (**D**) Left lateral ventricular choroid plexitis (white arrow).

**Figure 3:**
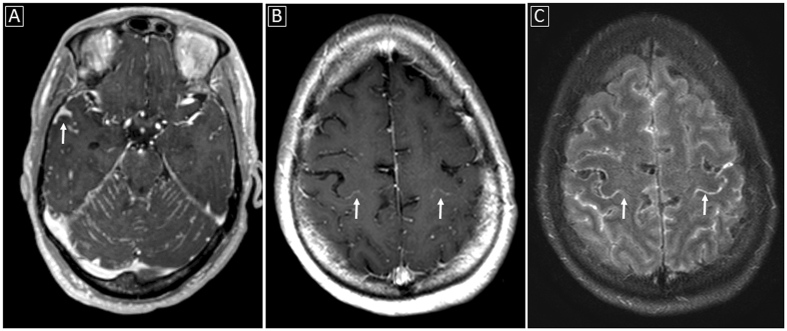
Examples of meningeal involvement in CNS cryptococcosis: (**A**) Abnormal meningeal enhancement along the cerebellar folia as well as along﻿ the sulci within the middle cranial fossae (white arrow) on enhanced T1-weighted image; (**B** and **C**) Sulcal meningeal enhancement especially delineating the central (white arrows) and postcentral sulci on enhanced T1-weighted and enhanced FLAIR images obtained near the convexity.

Imaging findings in the three patient groups are summarized in Table 2. Examples of the imaging findings are also shown in Figs 1, 2 and 3. While both HIV+ and HIV− patients with documented CM showed relatively similar rates of enhancing basal ganglia lesions (p-value = 0.72), non-enhancing basal ganglia prominent perivascular spaces (p-value = 0.74) and meningeal enhancement (p-value = 0.71), HIV− patients showed higher rates of enhancing parenchymal lesions (cryptococcomas) although not statistically significant (48.9% versus 27.3%, p-value = 0.31), and higher rates of ventricular manifestations including choroid plexitis (28.9% versus 0%, p-value = 0.051), ependymitis (35.6% versus 0%, p-value = 0.024) and hydrocephalus (51.1% versus 9.1%, p-value = 0.016).

**Table 2:** Summary of Imaging Findings in HIV− and HIV + subjects.

Parameters	HIV−patients with documented CNS cryptococcosis (n = 45)	HIV−patients with pulmonary nut no CNS cryptococcosis (n = 5)	HIV + patients with documented CNS cryptococcosis (n = 11)
Enhancing basal ganglia lesions	13 (28.9%)	0 (0%)	4 (36.4%)
Non-enhancing basal ganglia lesions (prominent perivascular spaces)	25 (55.6%)	1 (20%)	5 (45.5%)
Other parenchymal enhancing lesions	22 (48.9%)	0 (0%)	3 (27.3%)
Ependymitis	16 (35.6%)	0 (0%)	0 (0%)
Choroid plexitis	13 (28.9%)	0 (0%)	0 (0%)
Meningeal (pial) enhancement	32 (71.1%)	0 (0%)	9 (81.8%)
Hydrocephalus	23 (51.1%)	0 (0%)	1 (9.1%)

Applying the generalized linear model (GLM) analysis with natural log(sCD27) as the y-variable, and considering the group and all seven imaging findings as x-variables, the best set of predictors was documented CM (vs. localized disease as a control), ependymitis and hydrocephalus with respective p-values of 0.034, 0.012 and <0.003. Interestingly, when only assessing patients with documented CM (eliminating group), and excluding prominent perivascular spaces (generally irreversible/potentially normal variant) and hydrocephalus (cannot be appropriately assessed in patients with ventricular shunts) from the list of x-variables, the best predictor of higher sCD27 levels was found to be ependymitis with p-value of 0.0017.

For natural log(NFL) values in patients and controls, on the other hand, the “best” set of predictors was found to be a history of CM (group) and hydrocephalus with respective p-values of 0.007 and 0.002. When evaluating the patient group separately and excluding prominent perivascular spaces and hydrocephalus, as above, choroid plexitis became the best predictor of higher NFL levels although this did not reach statistical significance (p-value = 0.056).

## Discussion

CNS cryptococcosis in previously healthy HIV− subjects accounts for almost a third of the cases in developed countries^3^ and is associated with significant (~30%) mortality despite optimal therapy^2, 4, 5^. MR Imaging of the brain is usually one of the earliest diagnostic examinations in this setting and many therapeutic decisions are based on the intracranial imaging findings.

**Figure 4:**
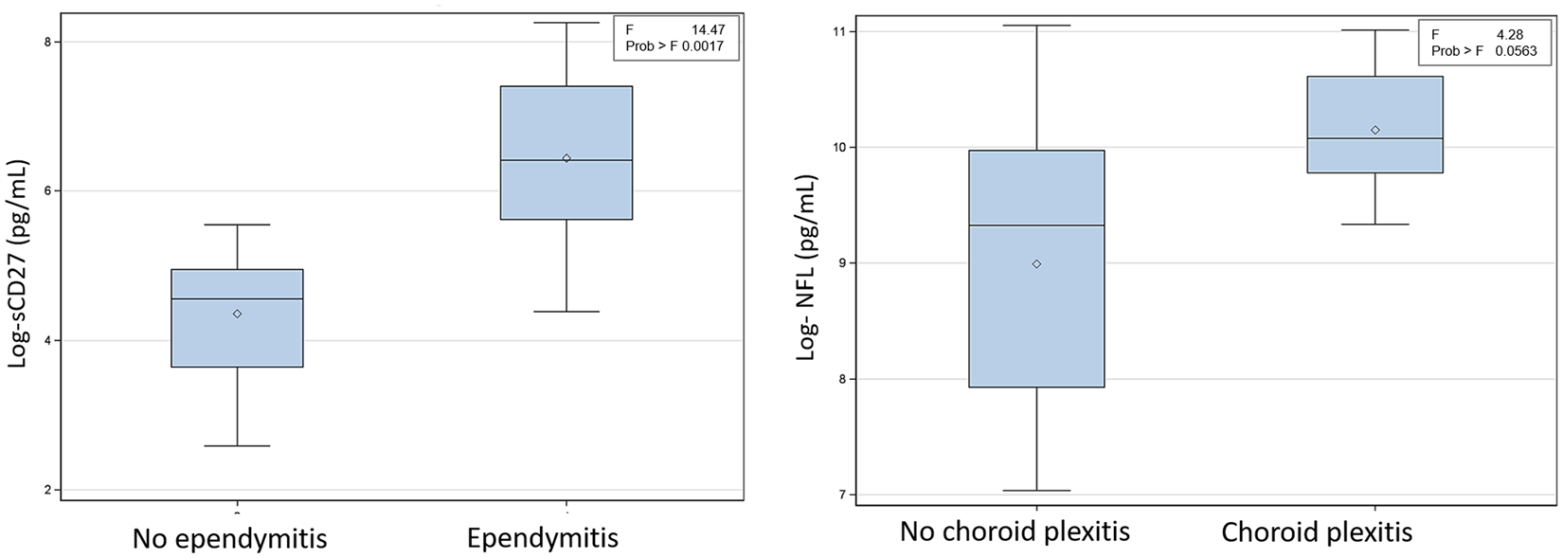
Box plots showing the distribution of log(sCD27) with respect to the presence of ependymitis and log(NFL) with respect to the presence of choroid plexitis.

In our patient population with CM, the HIV− group showed ventricular manifestations including choroid plexitis and ependymitis, neither of which seen in the HIV+ subjects. We believe those findings reflect a post-infectious inflammatory response syndrome (PIIRS) rather than microbiological failure since the majority of the patients had negative fungal CSF cultures. Those inflammatory changes have been previously seen in association with elevated levels of CSF NFL, a marker of axonal damage, consistent with ongoing neurological damage^5^. Similarly, sCD27 levels have been recently shown to best predict the presence of intrathecal T-cell mediated inflammation compared to other biomarkers^27^ and suppression of sCD27 has been associated with clinical improvement in CNS cryptococcal disease^6^. Therefore, to better gauge the pathophysiology of this inflammatory reaction using non-invasive biomarkers, we used MRI to identify the imaging findings most related to elevations in NFL and sCD27 levels in the CSF. Using a GLM analysis with imaging variables as x-variables and cytokine levels as y-variables, ependymitis and hydrocephalus were the strongest predictors of high log(sCD27) levels in the presence of symptomatic CM disease while hydrocephalus and a history of overt CM best predicted higher log(NFL) levels. However, since hydrocephalus cannot be appropriately evaluated post shunting and since prominent basal ganglia perivascular spaces are generally irreversible, and could sometimes reflect a normal variant, we performed a second GLM analysis excluding the two factors as potential predictors (x-variables). This assessment would potentially apply for patients being followed up after shunt insertion. In this analysis we found ependymitis to be the best predictor of higher sCD27 levels and choroid plexitis to be the best predictor of higher NFL levels (Fig. 4). These results suggest the importance of ependymal and choroid inflammatory changes as disease biomarkers in HIV− patients with clinical deterioration, of whom approximately 30% eventually succumb to the disease^28^.

Besides showing higher rates of ventricular manifestations, more HIV− patients presented with hydrocephalus (51.1%) compared to HIV+ patients (9.1%). The reason for this discrepancy in imaging findings between HIV+ and HIV− patients could potentially reflect a different mechanism of CNS spread although a more likely explanation is a difference in the immune reaction induced at the site of spread, in this case the blood-CSF barrier. While T-lymphocyte defects in HIV+ patients may explain vulnerability to cryptococcal (and other opportunistic) infections, the mechanism of susceptibility to CM in the HIV− population is not fully understood. A recently published detailed immunological study of HIV− infected patients with normal CD4 counts and refractory CM demonstrated that rather than showing T-cell immunity deficits, there was strong intrathecal expansion and activation of cells of both the innate and adaptive immunity including HLA-DR + CD4 + and CD8 + T-cells and NK cells^5^. Despite effective microbiological control, the expanded CSF T-cells included a majority of cryptococcal-antigen specific CD4 + T-cells, expressing high levels of IFN-γ, a pro-inflammatory cytokine. At the same time, while macrophage recruitment appeared intact, M2 macrophage polarization resulted in poor phagocytosis of fungal cells^5^. The ventricular involvement in HIV− subjects with CM is probably related to the mechanism of pathogen spread to the brain. As the interface between the CSF and systemic circulation, the choroid plexus is suggested as an important portal for entry of *Cryptococcus*. Anatomically, the papillary fronds of the choroid plexus protrude into the ventricles and have an epithelial lining that is continuous with the ependymal surface^29^. This could explain the co-occurrence of choroid plexitis and ependymitis in our HIV− patient population. Historically, a similar ependymal reaction has been reported experimentally in reaction to cryptococcal polysaccharide^30^. In patients, the ependymal reaction may be happening because of release of cellular material after treatment with antifungals. Along the same lines, higher rates of hydrocephalus in the HIV− population upon presentation can also be potentially explained by the inflammatory exudates obstructing CSF drainage at the level of the arachnoid granulations.

The management of cryptococcal meningitis in HIV− patients is not straightforward, and controlling the ensuing PIIRS can be very challenging. One suggested treatment approach is the use of corticosteroids (once microbiological clearance is documented by negative CSF cultures)^5, 31^ although the effectiveness and cost-benefit ratio remain debatable^31–34^. Irrespective of the treatment strategy, however, gauging treatment for PIIRS requires diagnostic certainty and repeated lumbar punctures can be prohibitive especially in clinically unstable patients. In addition, research markers such as sCD27 and NFL may not be readily available in most centers. Our study suggests enhanced brain MRI as a surrogate non-invasive imaging approach for following up HIV− patients with cryptococcal meningitis, which might prove to be of use in specific clinical scenarios. Besides commonly recognizable prognostic features such as hydrocephalus, increased lesion load or worsening meningeal enhancement, ependymitis and choroid plexitis appear to be especially reflective of immune activation patterns and could guide treatment in complex follow-up cases.

Limitations of our study include a small number of HIV+ patients, as well a slightly heterogeneous HIV− patient population as far as CSF antigen titers, microbiological control and treatment. We did however exclude HIV− patients who had received immunosuppressive regimens in order to better control for patient heterogeneity. Another limitation is possible referral bias with more severe cases evaluated in this cohort, which makes our results not fully applicable to all HIV− subjects with cryptococcal meningitis. However, this is exactly the cohort of subjects that would benefit most from our findings considering the inherent high morbidity and mortality figures associated with severe disease. In addition, we were not able correlate MRI findings to clinical outcome because of the few numbers of adverse events (1 death) in the HIV− cohort, perhaps due to the routine use of corticosteroids and immunosuppressive therapy in refractory cases. Larger validation cohorts may thus be required for further validation.

Finally, even though choroid plexitis and ependymitis are not specific for cryptococcal CNS involvement (also seen with other infectious and noninfectious processes such as tuberculosis, cytomegalovirus, nocardiosis, toxoplasmosis, Wegener’s granulomatosis, sarcoidosis, lymphoma and germinoma^35–41^), in the correct clinical setting, their presence on imaging should raise the suspicion for CM in HIV− patients. After diagnosis, such findings may provide an effective and readily available biomarker of inflammation in the setting of microbiological control that could help manage the subset of patients with clinical deterioration.

## Methods

### Subjects

HIV− subjects were participants in an observational cohort examining the host genetics and immunology of cryptococcal disease in previously healthy, non-HIV infected adults. HIV+ subjects were participants in a prospective study of HIV+ persons with CD4 counts <100 cells/µL who were naïve to ART and were starting ART at study entry. Written informed consent was obtained from all subjects. All experimental protocols were approved by the research ethics committee and Institutional Review Board of the NIAID (NIH). All methods were carried out in accordance with relevant guidelines and regulations.

A total of 56 patients diagnosed with CM were evaluated. Forty five patients were previously healthy HIV− and 11 were HIV+. In addition, 5 HIV− patients with pulmonary but no CM (negative cryptococcal antigen in the CSF) were evaluated with imaging and CSF cytokine levels.

Treatment information for antifungals (either Amphotericin B or Fluconazole), antiretroviral therapy and steroids at the time of scanning was collected for both the HIV+ and HIV− groups. In addition, CSF cryptococcal antigen titers and culture results for *C. neoformans* were recorded. In the HIV+ group, CD4 cell counts and plasma viral loads were measured at the time of imaging.

### MR Imaging Acquisition

MR scans were either performed at NIH using 1.5T or 3T Philips MR scanner or on outside basis using a variety of different scanners. All the scans included at least T1, T2, FLAIR, diffusion weighted and enhanced T1 weighted scans. Scans at NIH included post contrast FLAIR images. Only scans with good diagnostic quality were included.

### Structural MRI abnormalities

Brain MRI findings were stratified into three main categories: parenchymal (Fig. 1), ventricular (Fig. 2) and meningeal (Fig. 3) abnormalities. Parenchymal abnormalities encompassed enhancing basal ganglia lesion(s), non-enhancing T2-bright basal ganglia lesion(s) (prominent perivascular spaces also known as Virchow-Robin spaces) and parenchymal enhancing lesion(s) (cryptococcomas) outside the basal ganglia. Ventricular abnormalities included abnormal ependymal enhancement of one or more ventricles (ependymitis), enlargement and abnormal enhancement of the choroid plexus(es) (choroid plexitis) and hydrocephalus. Meningeal involvement mainly described abnormal pial enhancement. For all patients, the scans upon presentation or earliest available enhanced MRI scans (from outside institutions) were evaluated. Patient’s medical records were reviewed for pertinent clinical information at the time of scanning.

### CSF collection and analysis

CSF levels of sCD27 and NFL were measured in a subset of the HIV− CM patients at presentation (n = 17), as well as in 5 patients with pulmonary but no documented CM. None of the 17 patients included in this analysis was being treated with corticosteroids at the time of CSF collection.

### Statistics

Imaging findings of HIV+ and previously healthy HIV− patients were evaluated using comparison of proportions of the seven main MRI characteristics (dichotomous variables): enhancing basal ganglia lesion(s), non-enhancing prominent perivascular spaces, other parenchymal enhancing lesions (outside the basal ganglia), ependymitis, choroid plexitis, pial meningeal enhancement and hydrocephalus. The comparisons were performed using fisher’s exact tests (two-sided).

Regression analysis of two CSF cytokines (sCD27 and NFL) and MR imaging characteristics were performed in 17 previously healthy HIV− patients with documented CM and 5 previously healthy HIV− patients with pulmonary but no documented CM. Since the distribution of the measured cytokines (sCD27 and NFL) was skewed, the distributions of their natural log-transform were evaluated and were found to be closer to normal. With the natural log transformation, statistical model assumptions were met. Hence, all subsequent statistical models were based on the log-transform values rather than the absolute values.

We explored the association between log-cytokine levels (sCD27 and NFL), and several factors including the group (CM versus no CM) and seven imaging characteristics: enhancing basal ganglia lesion(s) (yes/no), non-enhancing prominent perivascular spaces (yes/no), other parenchymal enhancing lesions (yes/no), ependymitis (yes/no), choroid plexitis (yes/no), pial enhancement (yes/no) and hydrocephalus (yes/no). A repeated-measures mixed model was applied with the log-cytokine level as the response variable (Y-variable) and the factors listed above as potential predictors. The same analysis was repeated in the group of patients with documented CM (n = 17) for each cytokine, using five rather than 7 variables (excluding non-enhancing prominent perivascular spaces and hydrocephalus).

### Data Availability

The datasets generated during and/or analyzed during the current study are available from the corresponding author on reasonable request.
